# The relationship between medical students’ interest in learning and their ability to solve mathematical problems: the chain-mediating role of teacher-student relationship and self-efficacy

**DOI:** 10.3389/fpsyg.2025.1531262

**Published:** 2025-03-12

**Authors:** Li Yang, Jingwen Cui, Yi Zhang

**Affiliations:** ^1^School of Management, Shandong Second Medical University, Weifang, Shandong, China; ^2^Faculty of Education, Qufu Normal University, Qufu, Shandong, China; ^3^School of Public Health, Shandong Second Medical University, Weifang, Shandong, China; ^4^Affiliated Hospital of Shandong Second Medical University, Weifang, Shandong, China

**Keywords:** medical students, mathematical problem-solving ability, learning interest, teacher-student relationship, self-efficacy

## Abstract

**Introduction:**

Although the impact of learning interest on academic performance has been extensively studied, the chain-mediated mechanism by which medical students’mathematics learning interest influences competence through teacher-student relationships and self-efficacy remains underexplored. Empirical evidence utilizing multi-mediation models to test indirect effects is particularly lacking.

**Methods:**

This study investigated 806 Chinese medical students, assessing problem-solving ability using PISA mathematics items and examining the chain-mediated pathway of teacher-student relationships and mathematics self-efficacy via structural equation modeling (SEM) and bias-corrected bootstrap methods. After controlling for major, grade, and residence.

**Results:**

The results demonstrated: (1) The direct effect of mathematics learning interest on problem-solving ability was non-significant (effect size = 0.0101, 95% CI [-0.0144, 0.0346]); (2) The independent mediating effect of teacher-student relationships was non-significant (effect size = 0.0083, 95% CI [-0.0114, 0.0196]); (3) The independent mediating effect of mathematics self-efficacy was significant (effect size = 0.0140, 95% CI [0.0003, 0.0286], contribution rate = 40.79%); (4) The chain-mediated pathway of teacher-student relationships → self-efficacy reached significance (effect size = 0.0020, 95% CI [0.0003, 0.0048], contribution rate = 5.68%). The total mediation effect accounted for 70.66% of the total effect.

**Discussion:**

These findings confirm that self-efficacy serves as the critical mechanism translating medical students’ mathematics interest into competence. We recommend enhancing self-efficacy through clinical scenario-based simulation tasks and stepwise training programs, providing theoretical foundations for reforming medical mathematics curricula.

## 1 Introduction

The escalating demands on medical practitioners have brought mathematical literacy into increasing focus within medical education as an indispensable competency for medical students. Contemporary medical education is no longer confined to traditional biomedical knowledge but has shifted toward emphasizing interdisciplinary competencies, particularly the cultivation of mathematical literacy. The growing need for precise and quantitative analysis in modern medicine underscores an urgent demand for professionals with advanced mathematical literacy in future healthcare landscapes (Anderson and Giordano, 2013). A society’s innovative capacity hinges on its ability to attract talent into Science, Technology, Engineering, Mathematics, and Medicine (STEMM) fields. Interdisciplinary professionals integrating medicine and mathematics are poised to play pivotal roles in the era of artificial intelligence (AI) (Stoeger et al., 2024). Equipped with robust medical expertise, clinical experience, and proficiency in mathematical analysis, data modeling, and algorithmic development, these individuals can extract actionable insights from vast medical datasets, develop accurate disease prediction models, and design personalized treatment plans (Dhillon et al., 2022). Driven by AI advancements, the demand for such interdisciplinary talent will continue to rise, positioning them as key drivers of healthcare innovation and optimization.

Problem-solving ability serves as a core manifestation of mathematical literacy. Prior research indicates that the formation of mathematical literacy originates in early education and is influenced by multiple factors, including parental attitudes toward mathematics, teacher support, mathematics self-efficacy, and learning interest (Cheung et al., 2024; Denner et al., 2019; Jung et al., 2023; Viljaranta et al., 2014). However, existing studies predominantly focus on preschool and K-12 populations (Denner et al., 2019; Fisher et al., 2012; Sankalaite et al., 2023), leaving a significant gap in understanding mathematical literacy among university students, particularly medical students. This oversight is critical, as medical practice increasingly relies on advanced mathematical skills—from clinical drug dosage calculations and epidemiological statistical analyses to algorithmic applications in medical imaging (Goldstein et al., 2024; Liu et al., 2023). Despite STEMM disciplines emphasizing the intersection of mathematics and medicine, empirical investigations into the mechanisms and influencing factors of mathematical competency development in medical education remain scarce.

Targeting medical students, this study addresses two key questions: (1) the current status of mathematical problem-solving ability among medical students; and (2) how learning interest, teacher-student relationships, and self-efficacy collectively influence mathematical competency through chain-mediated effects. By shifting the research focus from younger learners to higher education populations within medical contexts, this study aims to bridge the gap in empirical literature on university-level mathematical literacy while providing evidence-based pathways for optimizing medical curricula.

### 1.1 Relationships among learning interest, teacher-student relationships, self-efficacy, and mathematical problem-solving ability

Research suggests that the reciprocal relationship between mathematical interest and competence may emerge as early as preschool, with learning interest shaping mathematical abilities during formative developmental stages (Fisher et al., 2012). Preschoolers’ mathematical interest, emotional skills, and pre-school mathematical competencies are intercorrelated (Doctoroff et al., 2016). In primary education, mathematical interest remains predictive of academic performance (Brumm and Rathgeb-Schnierer, 2023), while middle school studies from a social-cognitive perspective confirm its enduring influence on achievement outcomes (Petersen and Hyde, 2017). By high school, mathematical interest fosters advanced cognitive strategies that enhance academic performance (Yu and Singh, 2018). However, given the highly specialized and practice-oriented nature of mathematical applications in medical education, the mechanisms through which learning interest influences medical students’ problem-solving abilities remain unexplored. Thus, we propose:

*Hypothesis 1 (H1):* Mathematics learning interest positively predicts medical students’ mathematical problem-solving ability in higher education.

Regarding teacher-student relationships, active classroom engagement and responsive teacher-student interactions have been shown to enhance educators’ job satisfaction and instructional efficacy, indirectly improving academic outcomes (Holzberger et al., 2013). We therefore posit:

*Hypothesis 2 (H2):* Teacher-student relationships positively predict mathematical problem-solving ability.

Self-efficacy frequently mediates learning outcomes. Empirical studies demonstrate its role in boosting classroom participation (Jung et al., 2023) and mathematical achievement (Yang et al., 2021), leading to:

*Hypothesis 3 (H3):* Mathematics self-efficacy positively predicts medical students’ problem-solving ability.

### 1.2 Interplay among learning interest, teacher-student relationships, and self-efficacy

The direct influence of learning interest on teacher-student relationships is well-documented. Positive emotional engagement in team settings facilitates collaborative interactions and task completion (Warwas et al., 2009), implying that intrinsic motivation and interest strengthen interpersonal dynamics. Consequently, heightened learning interest fosters supportive teacher-student relationships (Huang et al., 2022), prompting:

*Hypothesis 4 (H4):* Mathematics learning interest positively predicts teacher-student relationships.

Learning interest drives self-efficacy development. From the Social Cognitive Career Theory (SCCT) perspective, self-efficacy shapes occupational attitudes and interests, with both constructs forming core components of professional identity (Armstrong and Vogel, 2009). Medical students’ learning interest enhances intrinsic motivation, thereby elevating academic engagement (Pelaccia and Viau, 2017). We hypothesize:

*Hypothesis 5 (H5):* Mathematics learning interest positively predicts self-efficacy.

Situational expectancy-value and control-value theories further indicate that teacher-student interactions modulate students’ perceived support, enabling educators to reinforce the value of learning interests and mitigate negative emotions in mathematics (Rubach et al., 2023). Supportive relationships amplify self-efficacy by fostering motivational resilience (Fasce et al., 2016), leading to:

*Hypothesis 6 (H6):* Teacher-student relationships positively predict self-efficacy.

### 1.3 Chain-mediated roles of teacher-student relationships and self-efficacy

In the context of medical education, teacher-student relationships and self-efficacy are theorized to mediate the link between learning interest and problem-solving ability. Positive teacher-student relationships create a low-stress, supportive learning environment that bolsters students’ confidence in mathematics. Analyses of PISA data reveal that such relationships predict mathematical achievement by enhancing interest and self-efficacy (You et al., 2021), with academic self-efficacy further driving performance through perseverance and goal-oriented behaviors (Tosto et al., 2016).

Self-efficacy critically shapes problem-solving competence by regulating learners’ emotional states [e.g., reducing anxiety (Wang et al., 2024)] and sustaining scientific curiosity (Cordero et al., 2010). Students with high self-efficacy exhibit greater persistence, employ metacognitive strategies, and explore diverse solutions when confronted with mathematical challenges (Ten Braak et al., 2022). Integrating these mechanisms, we propose:

*Hypothesis 7 (H7):* Teacher-student relationships mediate the effect of mathematics learning interest on problem-solving ability.

*Hypothesis 8 (H8):* Self-efficacy mediates the effect of mathematics learning interest on problem-solving ability.

*Hypothesis 9 (H9):* A chain-mediated pathway (learning interest → teacher-student relationships → self-efficacy) explains the relationship between learning interest and problem-solving ability.

## 2 Materials and methods

### 2.1 Participants

This study employed a randomized sampling method to distribute anonymous questionnaires at a medical university in Shandong Province, China. A total of 853 questionnaires were issued, with 806 valid responses retained, yielding a valid response rate of 94.49%. The final sample comprised 288 male students (35.7%) and 518 female students (64.3%). In terms of academic standing, 752 participants were first-year students (93.3%) and 54 were second-year students (6.7%).

### 2.2 Measures

This study employed a self-designed questionnaire consisting of two parts: a mathematical problem-solving ability test and a survey on factors influencing medical students’ mathematical competence. The ability test section included four publicly available PISA mathematical literacy test questions (e.g., shopping discount calculations and transportation route optimization). Questions 1–2 were selected from “occupational context” items to assess multi-step calculations and quantitative reasoning, while questions 3–4 were drawn from “social context” items requiring mathematical modeling to solve complex problems. All questions were categorized as PISA Level 4–5 (top 20% proficiency level for 15-year-olds). Scoring followed a binary system: fully correct answers received 0.5 points, with incorrect or incomplete responses awarded zero points. Questionnaire validity was ensured through dual verification, with item content rigorously aligned with the PISA “mathematization processes” competency framework.

The survey on factors influencing medical students’ mathematical problem-solving ability comprised 22 items adapted from existing literature. Part 1 collected demographic information (gender, grade, residence, and major) through four questions. Part 2 measured three dimensions of mathematical competence—learning interest, self-efficacy, and teacher-student relationships—using 18 items. Each item adopted a 5-point Likert scale, scored from 0.5 to 2.5 points to enhance response differentiation (Bu et al., 2023).

### 2.3 Reliability and validity of the questionnaire

To reduce potential response bias in self-reported data, this study implemented three measures: anonymous responses, randomized item sequencing, and attention-check questions (reverse-scored items), leading to the exclusion of 5.5% invalid questionnaires. Reliability analysis demonstrated strong internal consistency, with an overall Cronbach’s *α* coefficient of 0.919. Subscale coefficients were 0.801 for self-efficacy, 0.855 for learning interest, and 0.876 for teacher-student relationships (mean α = 0.844). Validity was verified through exploratory factor analysis (EFA). The Kaiser-Meyer-Olkin (KMO) measure reached 0.916, and Bartlett’s test of sphericity yielded *χ*^2^ = 11,246.7 (*p* < 0.001), confirming sampling adequacy. The cumulative variance explained was 66.29%, with factor loadings aligning closely with the predefined dimensions: Factor 1 (self-efficacy: loadings 0.72–0.89) explained 30.53% variance; Factor 2 (teacher-student relationships: loadings 0.65–0.83) accounted for 13.08%; Factor 3 (learning interest: loadings 0.61–0.79) contributed 9.04%. The remaining four factors each explained <5% variance (reflecting measurement error variability), but all cross-loadings remained below 0.40, confirming the structural validity of the hypothesized three-dimensional framework and the questionnaire’s robust psychometric quality.

### 2.4 Statistical analysis

Data analysis was conducted using SPSS v29.0 for descriptive statistics and Spearman’s rank-order correlation with a two-tailed significance threshold of *α* = 0.05. A serial mediation model was constructed via Model 6 in Hayes’ PROCESS macro v4.2 for SPSS. To evaluate the practical significance of findings, this study reported standardized regression coefficients and 95% confidence intervals (CIs). Additionally, Cohen’s *d* effect sizes were calculated, interpreted using Cohen’s (1988) heuristic benchmarks: *d* = 0.20–0.50 (small), 0.50–0.80 (medium), and > 0.80 (large). Notably, Cohen emphasized these thresholds as “conventional reference points,” and recent scholarship has cautioned against their uncritical application across contexts (Panjeh et al., 2023).

## 3 Results

### 3.1 CMB test

Common method bias (CMB) was tested using Harman’s single-factor test. Results showed 7 factors with eigenvalues greater than 1, with the first factor explaining 30.529% of variance—below the 40% critical threshold—indicating no significant common method bias. Potential biases (e.g., systematically inflated self-efficacy scores or elevated correlations due to uniform scoring formats) were mitigated by: (1) combining Likert scales with objective calculation tasks, (2) including reverse-scored items, and (3) enforcing anonymous responses. While CMB might modestly inflate correlation magnitudes, the clear differentiation of factor loadings and strong alignment between empirical data and theoretical dimensions support the robustness of core conclusions.

### 3.2 Distribution of the surveyed sample and variable control

In the surveyed sample, grade level, geographic residence, and academic major were included as control variables in subsequent regression and mediation analyses. Gender distribution showed 35.73% male and 64.27% female participants, with a higher proportion of females. First-year undergraduates accounted for 93.30% of the sample, reflecting a deliberate focus on first-year medical students. Geographic residence distribution was predominantly rural (335 participants), followed by cities (273 participants) and towns (198 participants). Academic majors were represented in descending order as Clinical Medicine, Biomedical Engineering, Medical Imaging, Health Administration, Anesthesiology, Emergency Management, Labor and Social Security, and Public Health. The skewed distributions of grade levels and majors (e.g., overrepresentation of first-year students and specific disciplines) may confound the relationship between academic interests and competencies due to variations in curricular intensity, requiring statistical control.

### 3.3 Significance of difference test

On the gender dimension, no significant differences were observed in scores for mathematical self-efficacy, mathematical learning interest, teacher-student relationships, or mathematical problem-solving ability. On the grade dimension, no significant differences were found for mathematical self-efficacy, mathematical learning interest, or teacher-student relationships, but first-year students scored higher than second-year students in mathematical problem-solving ability (*p* < 0.05). On the residence dimension, no significant differences emerged for mathematical self-efficacy, but medical students from towns scored higher than those from cities and rural areas in mathematical learning interest (*p* < 0.05), while urban medical students scored higher than those from towns and rural areas in teacher-student relationships (*p* < 0.05). On the major dimension, no significant differences existed for mathematical self-efficacy, but Emergency Management majors outperformed other disciplines in mathematical learning interest and teacher-student relationships (*p* < 0.05), and Anesthesiology majors scored higher than others in mathematical problem-solving ability (*p* < 0.05) (see Table 1).

**Table 1:** Results of significance of difference test.

Variables	Mathematical self-efficacy				Interest in mathematics learning				Teacher-student relationship				Mathematical problem solving ability			
	*M*	SD	*t* (*F*)	*P*	*M*	SD	*t* (*F*)	*P*	*M*	SD	*t* (*F*)	*P*	*M*	SD	*t* (*F*)	*P*
Gender			0.41	0.52			0.07	0.79			1.49	0.22			0.57	0.45
Female	11.13	2.41			10.26	2.54			12.76	1.81			1.36	0.71		
Male	11.25	2.79			10.21	2.96			12.58	2.14			1.40	0.73		
Grade			0.10	0.75			0.01	0.92			0.30	0.58			6.96	<0.05
Freshman	11.16	2.55			10.24	2.69			12.70	1.93			1.39	0.71		
Sophomore	11.28	2.54			10.28	2.77			12.56	2.03			1.13	0.78		
Residence			0.13	0.89			3.54	<0.05			3.20	<0.05			1.83	0.16
Urban	11.22	2.66			9.91	2.86			12.93	1.93			1.34	0.74		
Township	11.20	2.30			10.54	2.61			12.60	1.80			1.46	0.70		
Rural	11.12	2.60			10.34	2.59			12.56	1.99			1.36	0.71		
Profession			1.11	0.35			3.60	<0.05			2.06	<0.05			3.36	<0.05
Clinical medicine	11.12	2.72			9.66	2.94			12.89	1.75			1.47	0.69		
Anesthesiology	10.83	2.87			10.10	2.65			12.93	1.70			1.57	0.56		
Medical imaging	10.97	2.52			10.29	2.69			12.66	2.20			1.41	0.68		
Public health	10.83	2.13			9.65	2.35			12.00	1.35			1.22	0.67		
Health service management	11.46	2.33			10.61	2.28			12.72	1.73			1.11	0.85		
Emergency management	12.19	2.32			11.42	2.55			13.35	1.57			1.31	0.79		
Labor security	11.49	2.43			10.46	2.01			12.80	2.50			1.17	0.78		
Biomedical engineering	11.18	2.43			10.63	2.69			12.43	2.06			1.35	0.71		

### 3.4 Correlation analysis

Normality tests (Kolmogorov–Smirnov: D = 0.135–0.324; Shapiro–Wilk: W = 0.750–0.949; all *p* < 0.001) revealed significant deviations from normal distribution across all variables, thus justifying the use of Spearman’s rank-order correlation analysis. Correlation analysis revealed significantly positive correlations between medical students’ mathematical problem-solving ability and self-efficacy, learning interest, and teacher-student relationships (see Table 2). Specifically, the correlation coefficients between problem-solving ability and self-efficacy, learning interest, and teacher-student relationships were 0.142, 0.113, and 0.132, respectively. While statistically significant (*p* < 0.01), the small magnitude of these coefficients may reflect the context-specific nature of medical mathematics and the overall low performance in problem-solving ability (mean score = 1.38), which likely limited the strength of associations. In contrast, stronger correlations were observed: self-efficacy correlated with learning interest (*r* = 0.629) and teacher-student relationships (*r* = 0.419), while learning interest correlated with teacher-student relationships (*r* = 0.429), all with *p* < 0.01.

**Table 2:** Results of correlation analysis.

Variables	Score (x̄ ± s)	Learning interest	Teacher-student relationship	Self-efficacy	Math problem solving ability
Learning interest	10.24 ± 2.69	1.000			
Teacher-student relationship	12.69 ± 1.93	0.429^**^	1.000		
self-efficacy	11.17 ± 2.55	0.629^**^	0.419^**^	1.000	
Mathematical problem solving ability	1.38 ± 0.717	0.113^**^	0.132^**^	0.142^**^	1.000

^**^ *p < 0.01.*

### 3.5 Mediation analysis

**Figure 1:**
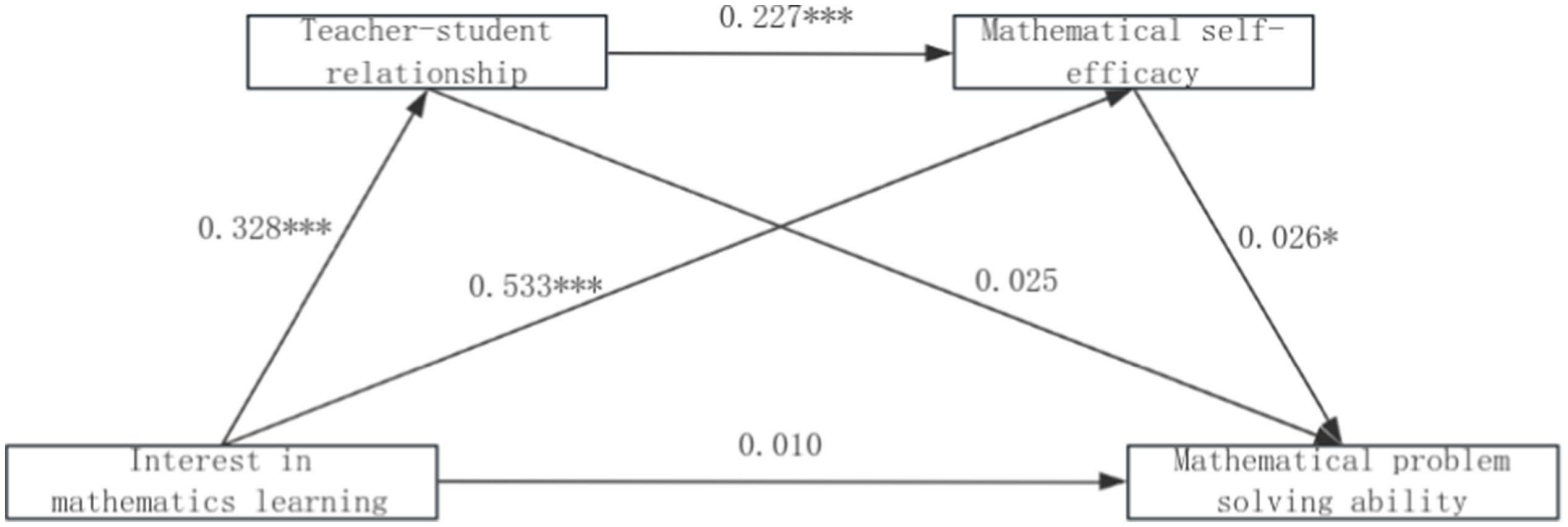
The mediating effect of self-efficacy and teacher-student relationship.

Using mathematical learning interest as the independent variable (*X*), mathematical problem-solving ability as the dependent variable (*Y*), and teacher-student relationships (*M1*) and mathematical self-efficacy (*M2*) as mediator variables, the analysis revealed the following: The total effect of learning interest on problem-solving ability was 0.034 (*p* < 0.001), with a 95% confidence interval of [0.016, 0.053] calculated via bootstrapping (5,000 samples), indicating that a 1-standard-deviation increase in learning interest corresponds to a 0.034-SD improvement in problem-solving ability (Cohen’s *d* = 0.12, small effect). The direct effect of learning interest on problem-solving ability was nonsignificant [*β* = 0.010, *p* > 0.05, 95% CI (−0.014, 0.035)]. For the teacher-student relationship (*M1*) mediation path (learning interest → teacher-student relationships → problem-solving ability), learning interest positively predicted teacher-student relationships (*β* = 0.328, *p* < 0.001), but the latter’s predictive effect on problem-solving ability was nonsignificant (*β* = 0.025, *p* > 0.05), yielding a nonsignificant indirect effect of 0.008 [95% CI (−0.011, 0.020)]. For the self-efficacy (*M2*) mediation path (learning interest → self-efficacy → problem-solving ability), learning interest positively predicted self-efficacy (*β* = 0.533, *p* < 0.001), and self-efficacy significantly predicted problem-solving ability (*β* = 0.026, *p* < 0.05), with an indirect effect of 0.014 [95% CI (0.0003, 0.029), Cohen’s *d* = 0.18, small effect]. For the chained mediation path (learning interest → teacher-student relationships → self-efficacy → problem-solving ability), teacher-student relationships positively predicted self-efficacy (*β* = 0.227, *p* < 0.001), with a chained indirect effect of 0.002 [95% CI (0.0003, 0.005), Cohen’s *d* = 0.04, small effect]. Detailed paths are shown in Figure 1, and results are summarized in Tables 3, 4.

**Table 3:** Results of mediation analysis.

Dependent variable	Independent variable	Bias regression coefficient				*R*	*R^2^*	*p*
		*β*	*SE*	*t*	*p*			
Teacher-student relationship	Constant	10.167	0.372	27.307	<0.001	0.464	0.216	<0.001
	Interest in mathematics learning	0.328	0.023	14.408	<0.001			
	Grade	−0.006	0.244	−0.027	0.979			
	Place of residence	−0.205	0.071	−2.896	0.004			
	Profession	−0.056	0.013	−4.441	<0.001			
Self-efficacy	Constant	2.986	0.584	5.114	<0.001	0.653	0.426	<0.001
	Teacher-student relationship	0.227	0.040	5.682	<0.001			
	Learning interest	0.533	0.029	18.511	<0.001			
	Grade	0.182	0.275	0.660	0.509			
	Place of residence	−0.101	0.080	−1.261	0.208			
	Profession	−0.019	0.014	−1.346	0.179			
Mathematical problem solving ability	Constant	0.949	0.215	4.408	<0.001	0.211	0.044	<0.001
	Teacher-student relationship	0.025	0.015	1.721	0.086			
	Self-efficacy	0.026	0.013	2.053	<0.05			
	Learning interest	0.010	0.012	0.808	0.419			
	Grade	−0.230	0.100	−2.296	<0.05			
	Place of residence	0.026	0.029	0.898	0.370			
	Profession	−0.014	0.005	−2.742	0.006			

**Table 4:** Results of significance test.

Path	Estimate	Boot S.E	Upper CI	Lower CI	Proportion
Ind1 Interest in learning → Relationship between teachers and students → ability to solve math problems	0.0083	0.0053	0.0196	−0.0114	24.19
Ind2 Learning interest → Self-efficacy → ability to solve math problems	0.0140	0.0072	0.0286	0.0003	40.79
Ind3 Learning interest → teacher-student relationship → Self-efficacy → ability to solve math problems	0.0020	0.0011	0.0048	0.0003	5.68
Total mediating effect	0.0243	0.0088	0.0439	0.0080	70.66
Direct effect	0.0101	0.0125	0.0346	−0.0144	29.34
Total effect	0.0344	0.0093	0.0528	0.0161	100

The total indirect effect (0.0243) accounted for 70.66% of the total effect. The nonsignificant standalone mediation via teacher-student relationships suggests that isolated improvements in teacher-student interactions have limited impact on medical mathematics competency unless integrated with clinical-contextualized teaching to activate cognitive support mechanisms. The self-efficacy mediation path was significant, explaining 40.79% of the total effect. The chained mediation path (teacher-student relationships → self-efficacy) explained 5.68%, highlighting the potential of leveraging “teacher-student support → self-efficacy” as an educational enhancer. Overall, self-efficacy is the key mediator linking learning interest to mathematical competency, whereas the weak effects of teacher-student relationships and the chained path suggest prioritizing self-efficacy over isolated relational improvements.

Academic major significantly negatively predicted both teacher-student relationships (*β* = −0.056, *p* < 0.001) and problem-solving ability (*β* = −0.014, *p* = 0.006), reflecting that high-intensity training in mainstream disciplines (e.g., Clinical Medicine) generally suppresses relational and mathematical development. However, disciplinary heterogeneity exists: Emergency Management majors exhibited superior teacher-student relationships and learning interest due to integrated mathematical modeling curricula, while Anesthesiology excelled in problem-solving ability owing to explicit mathematical applications (e.g., pharmacokinetic calculations). These findings indicate that the overall negative trend across medical disciplines may be moderated by subject-specific differences (see Table 3).

## 4 Discussion

### 4.1 The effects of students’ mathematical learning interest, teacher-student relationships, and self-efficacy on their mathematical problem-solving ability

This study found that Hypotheses 1 and 2 were unsupported, while Hypotheses 3, 4, 5, and 6 were supported—that is, neither college students’ mathematical learning interest nor teacher-student relationships directly predicted mathematical problem-solving ability. On one hand, interest reflects a trait-like preference for work activities, environments, or outcomes, which drives goal-oriented behaviors (Hyland et al., 2022). When students exhibit strong interest in mathematics, they are more likely to engage in proactive learning, exploration, and conceptual understanding, fostering not only deeper knowledge acquisition but also innovative thinking in problem-solving. Prior research indicates that interest exerts a stronger influence on mathematical performance compared to physics, chemistry, or biology (Jansen et al., 2016). Conversely, students lacking mathematical interest often struggle with motivation, leading to difficulties, disengagement, and reduced learning efficacy (Merrick and Maher, 2009). However, this study’s focus on medical students introduces a critical distinction: mathematics in medical curricula serves as a tool rather than a core discipline, potentially diluting interest’s direct effects due to confounding from professionally oriented motivations (Machalow et al., 2022). After controlling for grade, residential environment, and major-related variables, the direct effect of mathematical interest on problem-solving ability became nonsignificant. While academic interest is known to positively influence achievement through enhanced effort and persistence—with stronger interest-achievement linkages in natural sciences (Jansen et al., 2016)—mathematics in medical education is often perceived as a “service discipline,” where clinical motivations may weaken its interest-ability association via motivational filtering (Sun et al., 2024). For example, medical students’ academic interest shows minimal impact on medical academic performance (Wu et al., 2019), and in psychology, initial personal interest fails to predict undergraduates’ grades (Harackiewicz et al., 2008). This study expands the scope of interest-achievement research, suggesting that disciplinary attributes may moderate interest-ability relationships, with implications for medical curriculum integration.

Additionally, aligned with the expectancy-value model, medical students’ mathematical motivations are dominated by career goal commitments. Even with positive teacher-student relationships, if students perceive mathematics as minimally relevant to future clinical practice, intrinsic motivation becomes suppressed by motivational filtering, blocking the direct translation of relational support into mathematical competency (Wu et al., 2020).

### 4.2 Mediating roles of teacher-student relationships and self-efficacy in the relationship between students’ interest in mathematics and problem-solving abilities

This study found that teacher-student relationships did not exhibit a significant independent mediating role in the relationship between college students’ mathematical learning interest and problem-solving ability, thus Hypothesis 7 was unsupported. While prior research indicates that learning interest significantly positively predicts teacher-student relationships (Kwok et al., 2022)—a bidirectional interaction where both participants influence each other’s cognition, emotions, motivations, and behaviors (Korthagen et al., 2014; Holen et al., 2018). Such relationships in medical education are predominantly centered on clinical skills rather than mathematics, likely weakening their mediating role in mathematical competency (Wei and Cheng, 2022). Academic achievement ultimately requires students’ own efforts; for instance, studies analyzing the mediating role of self-efficacy in the relationship between proactive personality and academic performance among college athletes found that proactive personality enhances grades only through self-efficacy (Li et al., 2022). This explains why teacher-student relationships alone cannot directly predict problem-solving ability and must operate via self-efficacy’s mediation.

The study confirmed that mathematical self-efficacy serves as a significant independent mediator between learning interest and problem-solving ability, supporting Hypothesis 8. Learning interest exerted a stronger indirect effect on problem-solving ability through self-efficacy alone compared to the chained mediation via teacher-student relationships. Strong mathematical self-efficacy correlates closely with problem-solving ability (Arens et al., 2022), as students with high self-efficacy typically possess superior mathematical thinking skills, foundational knowledge, confidence (Guo et al., 2023), well-being (Datu and Mateo, 2020), and academic efficiency. This finding identifies a low-cost intervention target for medical education: short-term cognitive-behavioral training to enhance self-efficacy may be more feasible and impactful than restructuring teacher-student dynamics (Ashraf et al., 2024).

The study also validated Hypothesis 9, demonstrating a chained mediation effect (learning interest → teacher-student relationships → self-efficacy → problem-solving ability). Strong learning interest fosters teacher-student interactions, which in turn boost self-efficacy—a critical predictor of academic achievement (Wei et al., 2022). Even in hybrid learning environments, teacher support remains pivotal for elevating self-efficacy (Weber and Harzer, 2022).

In summary, medical students’ learning interest enhances problem-solving ability exclusively through self-efficacy, with both the independent mediation of self-efficacy (*β* = 0.014, 95% CI [0.0003, 0.029]) and the chained mediation (teacher-student relationships → self-efficacy) exerting significant positive effects. By focusing on medical students, this study expands the scope of research on learning interest and academic performance, revealing that mathematical learning interest cannot directly improve problem-solving ability but requires the amplification of mathematical self-efficacy.

## 5 Limitations and directions for future research

First, this study focused on medical college students, a group with distinct professional orientations that may inherently exhibit lower mathematical learning interest. Medical curricula prioritize biomedical knowledge and clinical skills, relegating mathematics to a peripheral role as a foundational discipline. This structural emphasis likely diminishes students’ awareness of mathematics’ importance, thereby dampening their interest and motivation. Notably, the sample was limited to medical schools in eastern urban comprehensive universities, excluding rural medical colleges and specialized vocational institutions, and failed to report participants’ socioeconomic status—factors that may constrain the generalizability of findings. Future research must expand the scope to include students from diverse majors, institutions (urban vs. rural, comprehensive vs. vocational), and socioeconomic backgrounds to strengthen external validity.

Second, the measurement of medical students’ mathematical problem-solving ability relied on PISA (Programme for International Student Assessment) test items, administered under timed conditions with explicit prohibitions against internet use. However, pre-existing familiarity with PISA questions among some students (due to prior exposure) and the mismatch between PISA’s general mathematics focus and medical students’ need for clinical-contextualized mathematical application may have introduced measurement bias. Future studies should develop tailored assessment tools that align with medical education’s unique demands, such as clinical pharmacokinetics or epidemiological modeling tasks, to more accurately capture medical students’ domain-specific mathematical competencies.

Third, as a cross-sectional study targeting first-year medical students at an academic transition point (post-high school), this research cannot address potential temporal dynamics in mathematical interest and ability. Longitudinal designs are critical to explore: (1) how mathematical interest and problem-solving skills evolve across medical training stages (e.g., from foundational coursework in Year 1 to clinical rotations in Year 5), and (2) whether interventions such as self-efficacy training programs or teacher-student relationship enhancements differentially impact clinical mathematical competency through randomized controlled trials.

## 6 Practical implications

Despite these limitations, our findings retain significant practical and academic value. College students with stronger mathematical problem-solving abilities within discipline-specific learning contexts consistently demonstrate higher mathematical literacy. This study empirically confirms that medical students’ mathematical learning interest enhances problem-solving ability by improving teacher-student relationships and strengthening self-efficacy, thereby fostering holistic mathematical literacy development. To operationalize these insights, we recommend that medical educators embed clinical mathematical cases (e.g., drug dosage calculations, epidemiological modeling) into curricula to activate instrumental interest, adopt task decomposition methods in teaching to progressively build self-efficacy, and establish a dual-mentor feedback mechanism (mathematics instructors assessing logical rigor, clinical mentors evaluating application validity) to synergistically reinforce the mediating role of teacher-student relationships. Additionally, policymakers should integrate self-efficacy into medical students’ core competency assessments and provide cognitive behavioral therapy (CBT)-based interventions for low-efficacy students, offering novel pathways for higher education mathematics reform and competency cultivation.

## Data Availability

The raw data supporting the conclusions of this article will be made available by the authors, without undue reservation.
